# Nonrandom Distribution of Azole Resistance across the Global Population of Aspergillus fumigatus

**DOI:** 10.1128/mBio.00392-19

**Published:** 2019-05-21

**Authors:** Thomas R. Sewell, Jianing Zhu, Johanna Rhodes, Ferry Hagen, Jacques F. Meis, Matthew C. Fisher, Thibaut Jombart

**Affiliations:** a MRC Centre for Global Infectious Disease Analysis, Department of Infectious Disease Epidemiology, Imperial College London, London, United Kingdom; b Department of Medical Mycology, Westerdijk Fungal Biodiversity Institute, Utrecht, The Netherlands; c Department of Medical Microbiology and Infectious Diseases, Canisius-Wilhelmina Hospital, Nijmegen, The Netherlands; d Centre of Expertise in Mycology Radboudumc/Canisius-Wilhelmina Ziekenhuis (CWZ), Nijmegen, The Netherlands; e London School of Hygiene & Tropical Medicine, London, United Kingdom; Vallabhbhai Patel Chest Institute; University of Nottingham; Centers for Disease Control and Prevention

**Keywords:** *Aspergillus fumigatus*, antifungal chemicals, azole resistance, fungal pathogen, fungicides, global distribution

## Abstract

The emergence of azole resistance in the pathogenic fungus Aspergillus fumigatus has continued to increase, with the dominant resistance mechanisms, consisting of a 34-nucleotide tandem repeat (TR_34_)/L98H and TR_46_/Y121F/T289A, now showing a structured global distribution. Using hierarchical clustering and multivariate analysis of 4,049 A. fumigatus isolates collected worldwide and genotyped at nine microsatellite loci using analysis of short tandem repeats of A. fumigatus (STR*Af*), we show that A. fumigatus can be subdivided into two broad clades and that *cyp51A* alleles TR_34_/L98H and TR_46_/Y121F/T289A are unevenly distributed across these two populations. Diversity indices show that azole-resistant isolates are genetically depauperate compared to their wild-type counterparts, compatible with selective sweeps accompanying the selection of beneficial mutations. Strikingly, we found that azole-resistant clones with identical microsatellite profiles were globally distributed and sourced from both clinical and environmental locations, confirming that azole resistance is an international public health concern. Our work provides a framework for the analysis of A. fumigatus isolates based on their microsatellite profile, which we have incorporated into a freely available, user-friendly R Shiny application (AfumID) that provides clinicians and researchers with a method for the fast, automated characterization of A. fumigatus genetic relatedness. Our study highlights the effect that azole drug resistance is having on the genetic diversity of A. fumigatus and emphasizes its global importance upon this medically important pathogenic fungus.

## Abstract (precis)

Azole drug resistance in the human-pathogenic fungus Aspergillus fumigatus continues to emerge, potentially leading to untreatable aspergillosis in immunosuppressed hosts. Two dominant, environmentally associated resistance mechanisms, which are thought to have evolved through selection by the agricultural application of azole fungicides, are now distributed globally. Understanding the effect that azole resistance is having on the genetic diversity and global population of A. fumigatus will help mitigate drug-resistant aspergillosis and maintain the azole class of fungicides for future use in both medicine and crop protection.

## INTRODUCTION

Aspergillus fumigatus is a ubiquitous, globally distributed ascomycete fungus with an ecological niche of decaying vegetation and soil (1). In recent years, questions regarding the ecology and evolution of A. fumigatus, motivated primarily by the organism's ability to infect immunocompromised hosts but also by the emergence of antifungal drug resistance, have been raised (2–4). A. fumigatus is an opportunistic pathogen causing a spectrum of respiratory illnesses, from asthma-like symptoms to invasive aspergillosis (IA), where mortality rates of 40 to 90% have been reported in immunocompromised patients (5–7). There is a limited arsenal of drugs available for the treatment and prophylaxis of A. fumigatus-related aspergillosis, with the azole antifungals itraconazole, posaconazole, and voriconazole acting as the frontline defense against this disease (8).

Predictably, resistance to azole antifungals has emerged, and in recent years there have been increasing reports of resistant A. fumigatus isolates recovered from patients with aspergillosis (9). Resistance to azole antifungals can evolve during azole therapy (10–12); however, the extensive use of agricultural azole compounds in the environment for crop protection has now been linked to the emergence of azole resistance in A. fumigatus populations (4, 13–16). The most commonly occurring mechanisms of azole resistance are alterations in *cyp51A* (*erg11*), the gene encoding sterol 14-demethylase (cytochrome P450 51A [CYP51A]), which is the target protein of azole antifungals (17–19). In A. fumigatus, resistance is most frequently caused by a tandem repeat (TR) in the promoter that is linked to single nucleotide polymorphisms (SNPs) in the coding sequence, exemplified by the widespread occurrence of two alleles, TR_34_/L98H and TR_46_/Y121F/T289A (9, 20). The most frequently observed genotype, TR_34_/L98H, consists of a 34-nucleotide tandem repeat (TR_34_) in the *cyp51A* promoter that upregulates mRNA expression and a leucine-to-histidine substitution in the coding sequence that most likely affects the interaction between the azole ligand and the protein heme cofactor (21–23). The less frequent but emerging genotype, TR_46_/Y121F/T289A, is also known to have elevated mRNA expression, and it has been shown that TR_46_ in association with two SNPs (Y121F/T289A) confers high levels of resistance to azoles, including voriconazole (24).

Delineating the genetic diversity and evolutionary life history of pathogenic fungi can help elucidate the epidemiology of infection, aid in the prophylaxis and treatment of the disease-causing pathogen, and further our understanding of antifungal resistance evolution (25). Multiple studies conducted in the past 30 years have used a range of molecular markers to help unravel the population genetics of A. fumigatus (14, 26–31). Findings have ranged from no genetic structure to multiple distinct clusters, with varying reproducibility being found across methodologies. One commonly recurring feature nonetheless has been the identification of two well-supported phylogenetic clades (9, 29, 32–35). Preliminary studies utilizing high-resolution whole-genome sequencing found that isolates harboring TR_34_/L98H were not randomly distributed across the phylogeny and that the two-clade population structure could be the result of azole-resistant genotypes perturbing the natural population structure of A. fumigatus via selective sweeps (9, 32).

Despite this, little is still known about the global distribution of azole resistance across the wider A. fumigatus metapopulation and the genetic relationships between azole-resistant isolates recovered from the environment and patients (36). Here, we amend these gaps in our knowledge by analyzing the genetic relatedness of a collection of 4,049 A. fumigatus isolates sampled worldwide and genotyped at 9 microsatellite loci within the context of the resistance genotypes TR_34_/L98H and TR_46_/Y121F/T289A. The methodology used in this study forms the basis of a user-friendly bioinformatic tool, AfumID, for clinicians and researchers to genetically characterize novel A. fumigatus isolates within the context of the wider population genetic structure of this fungus.

## RESULTS

### Genetic clustering of A. fumigatus.

Of the 4,049 A. fumigatus isolates, a total of 385 alleles were recorded across the nine microsatellite loci analyzed. All loci were polymorphic, with the number of alleles per locus ranging from 25 to 101. Average Nei's gene diversity for all loci was 0.87, varying between 0.76 and 0.97 for individual loci. There were 2,293 represented multilocus genotypes (MLGs).

Analysis of Bruvo’s distances, to investigate the genetic relationship between isolates, showed no significant bootstrap support for any major nodes (>70%) but some support for nodes connecting a small number of isolates (see Fig. S4 in the supplemental material). A predominantly distinct subset of isolates did form a small, well-supported clade, and these isolates appeared to be fundamentally different from the majority of the other isolates.

**FIG 1:**
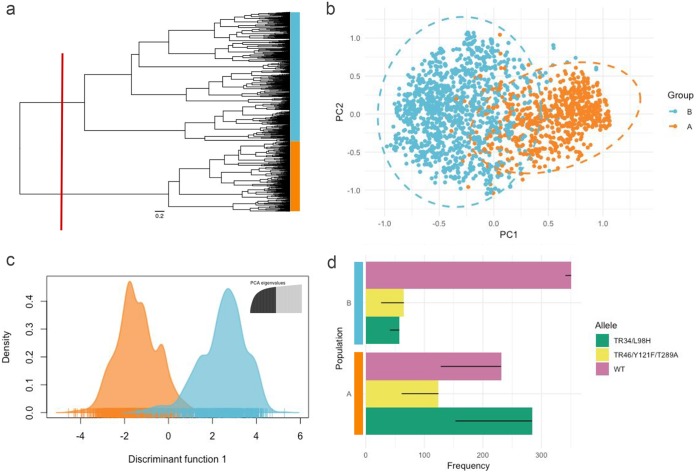
Genetic clustering and population differentiation of 4,049 Aspergillus fumigatus isolates genotyped at nine microsatellite loci. (a) Dendrogram representing the hierarchical clustering of isolates by Ward’s method. The relationship between isolates was determined using Bruvo’s distance. The red line indicates how the tree was divided for population definition. (b) Principal-coordinate analysis of Bruvo’s genetic distances between 4,049 A. fumigatus isolates factorially partitioned using populations based on prior hierarchical clustering. (c) Individual density plot from a modified discriminant analysis of principal components (DAPC) representing the first discriminant function. Populations A and B were discriminated by prior hierarchical clustering. (d) Bar plots illustrating the division of CYP51A alleles (WT, TR_34_/L98H, TR_46_/Y121F/T289A) across the two predicted populations. Thin black bars represent frequency changes after clone correction.

Hierarchical clustering of Bruvo’s distances by Ward’s method identified two broadly divergent clades, with 1,003 isolates being assigned to clade A and 1,781 being assigned to clade B (Fig. 1a). Principal-coordinate analysis (PCoA) on Bruvo’s distances further confirmed the existence of these two clusters when visualized, with moderate overlap on the first two PCoA axes, which accounted for the majority of the genetic diversity in the data (46.78% and 34.24% of the total variance, respectively), being seen (Fig. 1b). A 2-dimensional kernel density overlaid on the PCoA scatter plot, used to highlight compactness within the two clusters, suggested the existence of additional, more loosely defined subclusters within clades A and B (Fig. S5). Discriminant analysis of principal components (DAPC) cross validation further confirmed that the two clades were indeed genetically distinct, with the rate of success in assigning isolates to their correct clade using only their allelic profiles being 97%, a rate far exceeding random expectations (Fig. 1c; Fig. S6). Allele richness, which here indicates variations in genetic diversity, differed between the two clades, with group A having 268 alleles and group B having 345 alleles.

The frequency of the TR_34_/L98 and TR_46_/Y121F/T289A resistance genotypes was not evenly distributed across the two clades both with and without a clone correction step (Pearson's chi-square test, χ^2^ = 205.39, degrees of freedom [df] = 2, and *P* < 2.2e−16 and χ^2^ = 152.67, df = 2, and *P* < 2.2e−16, respectively; Fig. S7). Clade A, which had 639 isolates with an annotated CYP51A allele, consisted of 44.4% TR_34_/L98H, 19.4% TR_46_/Y121F/T289A, and 36.2% wild-type (WT) isolates. Clade B, which had 473 isolates with an annotated CYP51A allele, consisted of 12.1% TR_34_/L98H, 13.7% TR_46_/Y121F/T289A, and 74.2% WT isolates (Fig. 1d).

### Genetic diversity of azole-resistant A. fumigatus isolates.

Allelic richness (the total number of MLGs) was lower in the TR_34_/L98H (*n* = 175) and TR_46_/Y121F/T289A (*n* = 74) groups than in the WT group (*n* = 439). As richness is influenced by sample size, the number of effective multilocus genotypes (eMLGs) was also analyzed; both resistant groups—TR_34_/L98H (*n* = 110) and TR_46_/Y121F/T289A (*n* = 74)—retained a lower number of eMLGs than the WT group (*n* = 164), despite rarefaction to the smallest sample size.

**FIG 2:**
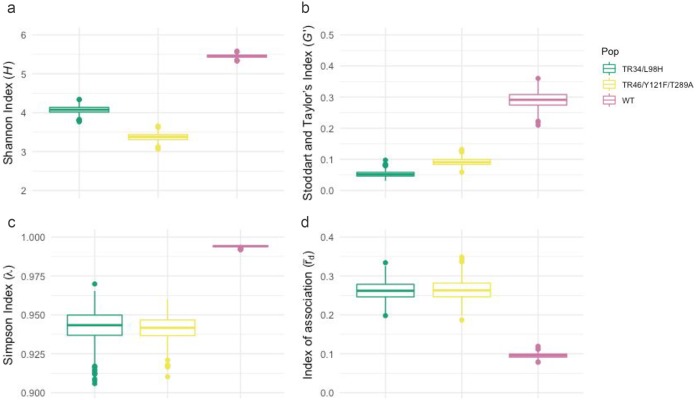
Genetic diversity indices of resistant Aspergillus fumigatus isolates harboring either the WT, TR_34_/L98H, or TR_46_/Y121F/T289A CYP51A allele. (a) Shannon-Wiener index of MLG diversity (*H*) (61); (b) the standardized Stoddart and Taylor’s index of MLG diversity (*G*′) (63); (c) Simpson’s index of MLG diversity (λ) (60); (d) index of association (${\bar{r}}_{d}$), which represents the clonal relationship of isolates (65, 66). Confidence intervals were generated with 1,000 bootstrap samples using the R package poppr. Pop, population.

According to all three diversity indices (Fig. 2a to c), the Shannon-Wiener diversity index (*H*; df = 2, *P* < 0.001), Simpson’s index (λ; df = 2, *P* < 0.001), and the standardized Stoddart and Taylor's index (*G*′; df = 2, *P* < 0.001), the resistant populations were significantly less diverse than the WT population (Fig. 2a to c). This held true with the introduction of a clone correction step, although, as expected, the diversity of populations with the TR_34_/L98H and TR_46_/Y121F/T289A genotypes did increase due to the reduction in the frequency of clonal genotypes (Fig. S8). There was a significant difference between the TR_34_/L98H and TR_46_/Y121F/T289A genotypes for *H* (df = 2, *P* < 0.001) but no significant difference for λ (df = 2, *P* = 0.268) and *G*′ (df = 2, *P* *=* 0.948). Isolates with the TR_34_/L98H and TR_46_/Y121F/T289A genotypes had a higher standardized index of association than the wild-type isolates, demonstrating a relationship among azole-resistant isolates more clonal than that found among wild-type isolates (df = 2, *P* < 0.001) (Fig. 2d).

### Distribution of azole-resistant multilocus genotypes.

**FIG 3:**
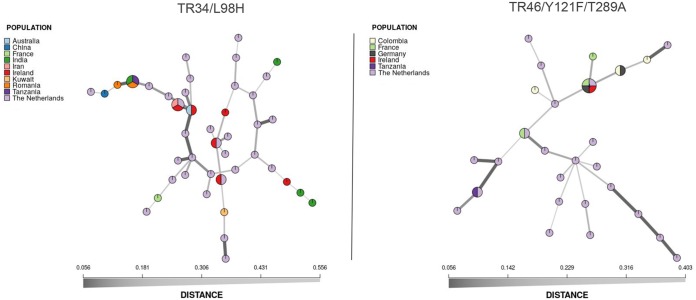
Minimum-spanning networks showing the geographic distribution and genetic relationship of Aspergillus fumigatus multilocus genotypes (clones) with either the TR_34_/L98H CYP51A allele or the TR_46_/Y121F/T289A CYP51A allele. The distance between MLGs is based on Bruvo’s genetic distances, which accounts for the stepwise mutation of microsatellite loci. Each node represents an MLG with two or more individuals. Nodes that are more closely related have darker and thicker edges, whereas nodes that are more distantly related have lighter and thinner edges.

**FIG 4:**
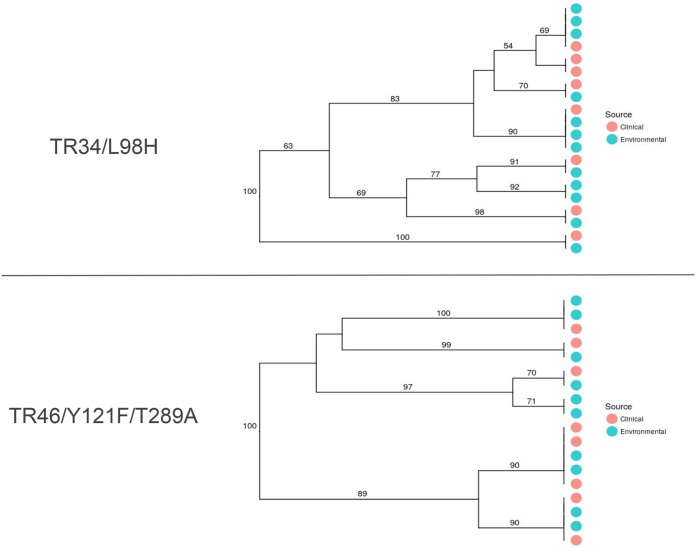
Neighbor-joining dendrograms for azole-resistant Aspergillus fumigatus clones from clinical or environmental sources with either the TR_34_/L98H or the TR_46_/Y121F/T289A allele. Each tip represents a resistant isolate that shares an MLG with two or more other isolates in the clone-corrected data set. Tip labels are shaded by source of isolation. Bootstrap values were generated using 1,000 samples.

There was no significant correlation between pairwise genetic and geographic distances for the TR_34_/L98H (*r*^2^ = 0.0299; *P* = 0.444), TR_46_/Y121F/T289A (*r*^2^ = 0.0476, *P* = 0.434), or WT (*r*^2^ = −0.0762, *P* = 0.729) genotype (Fig. S9). Therefore, there were no spatially structured patterns of genetic variation (isolation by distance [IBD]) for either the resistant or the WT isolates. Resistant MLGs were globally distributed, spanning multiple countries and multiple continents (Fig. 3). For example, isolates harboring TR_34_/L98H with identical microsatellite profiles (MLGs) were collected in Tanzania (Africa), Romania (Europe), and India (Asia). Additionally, TR_46_/Y121F/T289A-containing isolates were also widely distributed, with one MLG being found in four European countries (France, Germany, Ireland, and The Netherlands) and another closely related MLG being found in both Germany (Europe) and Colombia (South America). Resistant isolates with matching MLGs were also distributed among clinical and environmental sources (Fig. 4). There were six occasions where TR_34_/L98H-containing isolates with matching MLGs were found in both environmental and clinical sources and five occasions where TR_46_/Y121F/T289A isolates were found in both sources.

There were 144 short tandem repeat of A. fumigatus (STR*Af*) genotype duplicates found in both environmental and clinical locations. Of the 999 replicated simulations, zero genotype duplicates were generated, showing that the chance that two STR*Af* genotypes would randomly occur in both a clinical location and an environmental location without a relationship is very low (*P* = 0.01) (Fig. S10).

## DISCUSSION

In recent years, the population genetic structure of Aspergillus fumigatus has been well studied by utilizing a range of molecular markers to elucidate the genetic diversity and life history of this ubiquitous human fungal pathogen (9, 26–29, 32, 33, 35). Nevertheless, our understanding of the emergence of azole antifungal resistance and the effect that this has had on the genetic diversity of contemporary A. fumigatus populations remains understudied. Here, we addressed these gaps in our knowledge by exploiting the vast global STR*Af* collection held at the Centre of Expertise in Mycology, Radboudumc/Canisius-Wilhelmina Ziekenhuis (CWZ), in Nijmegen, The Netherlands. First, we show that the collection of 4,049 A. fumigatus strains can be clustered into two broadly defined populations and that two known resistance genotypes, TR_34_/L98H and TR_46_/Y121F/T289A, are unevenly distributed across these two populations. Second, resistant isolates harboring either TR_34_/L98H or TR_46_/Y121F/T289A are genetically depauperate compared to the wild types. Third, resistant clones are globally distributed and found in both environmental and clinical settings. Finally, we present AfumID, an R Shiny application that allows users to easily explore the genetic relationship of novel STR*Af* genotypes in relation to the full STR*Af* data set.

There has never been a definitive answer to the population differentiation of A. fumigatus, with multiple studies reporting a range of optimal population subdivisions (9, 26, 28, 29, 32). Here, we have shown that although there is no apparent clustering with regard to raw Bruvo’s distances, when isolates are hierarchically clustered using Ward’s method, which minimizes the total within-cluster variance and maximizes the between-cluster variance, two broad clades are found. Differentiation of these two clades was reinforced by a modified DAPC analysis, which revealed a detectable difference in allele frequencies when populations were discriminated by prior hierarchical clustering. Studies exploiting multilocus sequence typing or genomic sequencing have similarly revealed the presence of a biclade population structure (9, 29, 32, 35), further substantiating our findings and advocating a robust deviation from panmixia. Moreover, our demonstration of the nonrandom distribution of resistance genotypes TR_34_/L98H and TR_46_/Y121F/T289A across the two populations suggests a genetic component restricting the resistance genotype to one of the two populations. Sexual crossing both within and between populations may provide more insight into the potential for gene flow across the metapopulation and the propensity for introgression of resistant alleles (36, 37).

The genetic background of isolates harboring either TR_34_/L98H or TR_46_/Y121F/T289A was less diverse than that of the nonresistant, wild-type isolates. All four indices exhibited a significant reduction in diversity, revealing a pattern consistent with the rapid selection of beneficial mutations and the clonal expansion of isolates with highly fit resistance genotypes (38). Recent genomic studies have observed an expansion of the TR_34_/L98H genotype in India (32, 39), and according to our findings, it would appear that this situation may be a common occurrence worldwide. Indeed, it is apparent that identical clonal A. fumigatus isolates harboring TR_34_/L98H and TR_46_/Y121F/T289A occur globally, evidence that large geographic distances are not a barrier to the potential distribution of this species. This further supports the idea that A. fumigatus is a truly global fungus with the potential to migrate across vast areas of land and sea by virtue of both environmental (passive) and anthropogenic (active) dispersal (26). Numerous country-based studies have now identified clinical and environmental azole-resistant isolates harboring the two main resistance genotypes (40), and many have been incorporated into the STR*Af* database used in this study. However, until now, understanding the relationship between these isolates was limited (26). Here, we distinctly show that many azole-resistant isolates with identical microsatellite profiles occur worldwide and present a globally important threat to the use and stewardship of this important class of drugs (3).

Our study has also shown there to a be recurrent relationship between environmental and clinical isolates. On multiple occasions, we have shown that azole-resistant A. fumigatus clones sharing the exact same STR*Af* genotype were sourced from both environmental and clinical locations, a pattern that we also show to be vanishingly unlikely to occur by chance alone. This finding strengthens previous research that has shown similar results (34, 41, 42). Moreover, considering our current understanding that A. fumigatus conidia are unlikely to be transferred from patient to patient and are therefore unlikely to transfer from patient to the environment, it is highly likely that isolates harboring either TR_34_/L98H or TR_46_/Y121F/T289A have been acquired from environmental sources (43).

Antifungal resistance is increasingly a global problem in both agricultural and health care settings, and the evolution of azole resistance in A. fumigatus presents a worrisome contribution to this precarious situation (2, 44). Understanding the manner by which way azole resistance alleles are being distributed both genetically and spatially will greatly enhance our knowledge of the evolution of resistance and may help to engineer a response to the continued emergence of aspergillosis. By incorporating our analysis into a user-friendly application, we have provided clinicians and researchers with a method for the fast, automated characterization of A. fumigatus isolates that will inform the epidemiological study of patients’ infections and inform drug stewardship decisions in health care settings for this increasingly important pathogen.

## MATERIALS AND METHODS

### Study system.

Short tandem repeat (STR) genotypes for the 4,049 isolates used in this study were obtained from the short tandem repeat of A. fumigatus (STR*Af*) barcoding databank for clinical and environmental A. fumigatus strains, which is based at the Centre of Expertise in Mycology, Radboudumc/Canisius-Wilhelmina Ziekenhuis (CWZ), in Nijmegen, The Netherlands (45). The collection spans 26 countries across four continents (see Fig. S1 in the supplemental material) and includes metadata on source environment (environmentally or clinically sourced) and the presence or absence of azole resistance genotypes TR_34_/L98H and TR_46_/Y121F/T289A, where available. All isolates in the database were barcoded using a panel of nine short tandem repeat (STR) loci (namely, STR*Af* 2A, 2B, 2C, 3A, 3B, 3C, 4A, 4B, and 4C), as previously described (46) and validated (47). All STRA*f* genotypes were generated at CWZ. Of the 4,049 isolates, a subset of 1,112 had been genotyped for either TR_34_/L98H, TR_46_/Y121F/T289A, or wild-type (WT) CYP51A.

10.1128/mBio.00392-19.1 FIG S1 World map displaying the global distribution of the STR*Af* isolates used in this study. Download FIG S1, PDF file, 0.06 MB. Copyright © 2019 Sewell et al. 2019 Sewell et al. This content is distributed under the terms of the Creative Commons Attribution 4.0 International license.

### Global genetic structure of A. fumigatus.

Due to the predominantly clonal reproductive strategy of A. fumigatus, we used a multivariate, distance-based clustering approach, as opposed to methods that rely on assumptions such as linkage equilibrium and panmixia, to investigate the presence of the population structure (48, 49). First, considering that pockets of oversampled clones can complicate the identification of structure (50), a clone correction step was performed using the package poppr (v2.8.0) (51) for R software (52). Clone correction was based on geographic origin, source environment, and resistance genotype, in which a single isolate per multilocus genotype (MLG) was represented for each of the three strata. A total of 2,784 isolates were retained following the clone correction step. Before analysis, a genotype accumulation curve was generated to confirm that the STR*Af* data set had the correct number of loci (power) to discriminate between individuals in a population (Fig. S2) (50).

10.1128/mBio.00392-19.2 FIG S2 Genotype accumulation curve. Download FIG S2, PDF file, 0.07 MB. Copyright © 2019 Sewell et al. 2019 Sewell et al. This content is distributed under the terms of the Creative Commons Attribution 4.0 International license.

Genetic distances between individual isolates were determined using Bruvo’s distance in the R package poppr, which utilizes a stepwise mutation model for microsatellite loci (53). Genetic distances were calculated with 1,000-sample bootstrap support. Pairwise distances were hierarchically clustered using Ward’s method with the R function hclust and visualized on an ultrametric dendrogram. Population definitions (*K*) were selected using the R function cutree, where, on inspection, the tree was parsimoniously divided into two broad clusters (*K *=* *2). A principal-coordinate analysis (PCoA) (54) of genetic distances between isolates was calculated using the R package ade4 (v1.7-11) (55), after ensuring that the distances were Euclidean using the Cailliez transformation (ade4) (56, 57). The first two principal axes of PCoA were retained and visualized using a scatter plot where the two identified clusters were overlaid.

To identify whether populations defined by Ward’s clustering were fundamentally different, a modified discriminant analysis of principal components (DAPC) (58) in which the PCoA axes were used as input variables was used. The resulting analysis identified linear combinations of the PCoA axes exhibiting the strongest discrimination between clusters. DAPC was carried out using the package adegenet (v2.1.1), using cross validation to select the optimal number of variables retained in the initial dimension reduction step.

An interactive, web-based tool, AfumID, using information generated from this analysis was created using the R package Shiny (v1.0.5) and can be accessed at http://afumid.shinyapps.io/afumID. AfumID allows users to input microsatellite genotypes, together with metadata, to receive a brief characterization of their isolate relative to a subset of the STR*Af* database (Fig. S3). AfumID outputs include a notification as to whether the STR*Af* profile has been previously found and to which of the two populations the input isolate belongs. Users can also browse the CWZ database and visualize their isolate’s position on the PCoA scatter plot.

10.1128/mBio.00392-19.3 FIG S3 Screen shot of the R Shiny tool AfumID. Download FIG S3, PDF file, 0.3 MB. Copyright © 2019 Sewell et al. 2019 Sewell et al. This content is distributed under the terms of the Creative Commons Attribution 4.0 International license.

10.1128/mBio.00392-19.4 FIG S4 Dendrogram based on Bruvo genetic distance. Download FIG S4, PDF file, 0.3 MB. Copyright © 2019 Sewell et al. 2019 Sewell et al. This content is distributed under the terms of the Creative Commons Attribution 4.0 International license.

10.1128/mBio.00392-19.5 FIG S5 PCoA scatter plot with kernel density estimation. Download FIG S5, PDF file, 0.1 MB. Copyright © 2019 Sewell et al. 2019 Sewell et al. This content is distributed under the terms of the Creative Commons Attribution 4.0 International license.

10.1128/mBio.00392-19.6 FIG S6 DAPC cross validation. Download FIG S6, PDF file, 0.06 MB. Copyright © 2019 Sewell et al. 2019 Sewell et al. This content is distributed under the terms of the Creative Commons Attribution 4.0 International license.

10.1128/mBio.00392-19.7 FIG S7 Association (mosaic) plot. Download FIG S7, PDF file, 0.08 MB. Copyright © 2019 Sewell et al. 2019 Sewell et al. This content is distributed under the terms of the Creative Commons Attribution 4.0 International license.

10.1128/mBio.00392-19.8 FIG S8 Clone-corrected genetic diversity indices of resistant Aspergillus fumigatus isolates harboring either the WT, TR_34_/L98H, or TR_46_/Y121F/T289A CYP51A allele. Download FIG S8, PDF file, 0.1 MB. Copyright © 2019 Sewell et al. 2019 Sewell et al. This content is distributed under the terms of the Creative Commons Attribution 4.0 International license.

10.1128/mBio.00392-19.9 FIG S9 Isolation-by-distance scatter plot illustrating the pairwise relationship between genetic distance (country-based *F*_ST_) and geographic distances (in kilometers). Download FIG S9, PDF file, 0.07 MB. Copyright © 2019 Sewell et al. 2019 Sewell et al. This content is distributed under the terms of the Creative Commons Attribution 4.0 International license.

10.1128/mBio.00392-19.10 FIG S10 Histogram of simulated genotypes. Download FIG S10, PDF file, 0.05 MB. Copyright © 2019 Sewell et al. 2019 Sewell et al. This content is distributed under the terms of the Creative Commons Attribution 4.0 International license.

### Genetic diversity of resistant A. fumigatus.

The frequency of resistance genotypes across the predicted populations (*K *=* *2) was calculated, and the significance of the association was determined using Pearson’s chi-square test in R. The association was visualized using an extended association plot in the R package vcd (v1.4-4) (59). The association of a resistance allele to a specific population was deemed significant when *P* was <0.05.

A subset of 1,112 isolates with an annotated CYP51A allele (TR_34_/L98H, TR_46_/Y121F/T289A, or WT) was retained to determine the genetic diversity of azole-resistant isolates, which was performed without previously generated population associations. Allelic richness (total number of MLGs), the number of effective multilocus genotypes (eMLGs), Simpson’s index (λ), the Shannon-Wiener diversity index (*H*), the standardized Stoddart and Taylor's index (*G*′), and the standardized index of association (${\bar{r}}_{d}$) were all calculated using the R package poppr. Rarefied MLG, given here as eMLG, is the number of MLGs for each group at the lowest sample size across all groups. Simpson’s index is a measure of MLG diversity that takes into account the number of different MLGs present, as well as the MLG abundance (60). Shannon-Wiener diversity indices measures genotypic diversity by combining both MLG richness and MLG evenness (61). The standardized Stoddart and Taylor's index measures genotypic diversity while taking into account sample sizes (62, 63). The standardized index of association has been widely used as a tool to detect clonal reproduction within populations (64–66) and is used here to measure the relatedness of resistant and wild-type A. fumigatus isolates. All indices were calculated with (*n* = 750) and without (*n* = 1112) a clone correction step, to determine the importance of clones and identify patterns of clonal expansion. Confidence intervals were generated using 1,000 bootstrap samples. An analysis of variance was used to compare the diversity indices of different resistance genotypes, and Tukey’s test was used to identify which genotypes differed significantly.

### Distribution of multidrug-resistant clones.

To examine the global distribution of drug-resistant clones, a subset of resistant isolates with identical MLGs and with at least two or more representative individuals was used to generate minimum-spanning networks (MSN) based on Bruvo’s distances, using the R package poppr. Each node of the MSN represents a single MLG that consists of two or more individuals. Nodes or MLGs that are more closely related have darker and thicker edges, whereas nodes that are more distantly related have lighter and thinner edges.

To test for spatial patterns of genetic variation via isolation by distance (IBD), a Mantel test was performed to elucidate an association between geographic distance and the genetic divergence of azole-resistant isolates (67). Pairwise geographic distances between countries were based on the great-circle distance according to the haversine formula, which was computed using the R package geosphere (v1.5-7) (68). A pairwise fixation index (*F*_ST_) between countries was calculated using the R package hierfstat (v0.04-22) (69) for the TR_34_/L98H, TR_46_/Y121F/T289A, and WT genotypes. A Mantel test was performed on the matrices of the represented pairwise *F*_ST_ and geographic distances using the R package ade4 and 999 permutations.

A nonparametric bootstrapped (1,000-sample) unweighted pair group method using average linkages (UPGMA) dendrogram was produced using Bruvo’s distances to highlight the relatedness of a subset of resistant isolates with identical MLGs sourced from clinical and environmental locales. We used a nonparametric, Monte Carlo test to assess whether the occurrence of isolates with identical MLGs in both environmental and clinical settings deviated significantly from random expectations. The number of observed genotype duplicates across both settings was used as a test statistic. To generate the expected distribution of the statistic, we simulated environmental and clinical genotypes from the groups’ allele frequencies using the function hybridize in the adegenet package. A total of 999 independent random replicates were used for the test. The *P* value was calculated as the proportion of simulations greater than or equal to the original test statistic.
