# Supplementary figures and images for: Nonrandom Distribution of Azole Resistance across the Global Population of Aspergillus fumigatus
Source: mBio. 2019 May 21;10(3):e00392-19. doi: 10.1128/mBio.00392-19 (PMC6529631; doi:10.1128/mBio.00392-19)

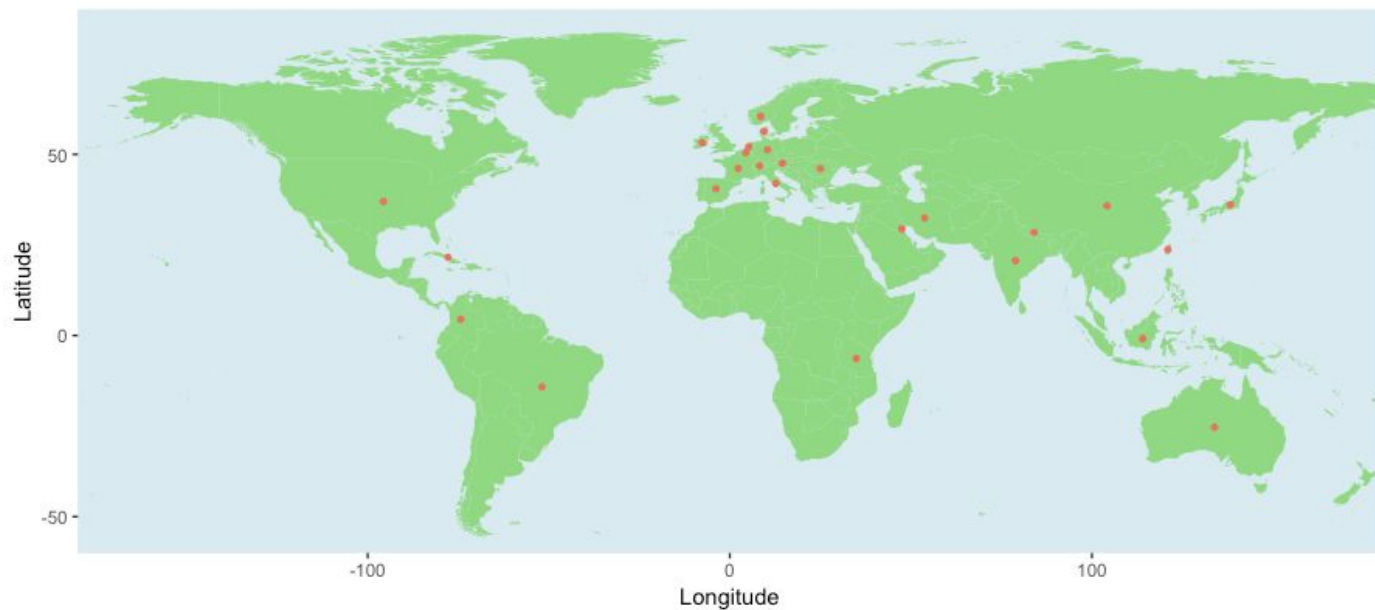

Fig S1: World map displaying the global distribution of the STRAf isolates used in this study

Supplement: FIG S1 [file mBio.00392-19-sf001.pdf]

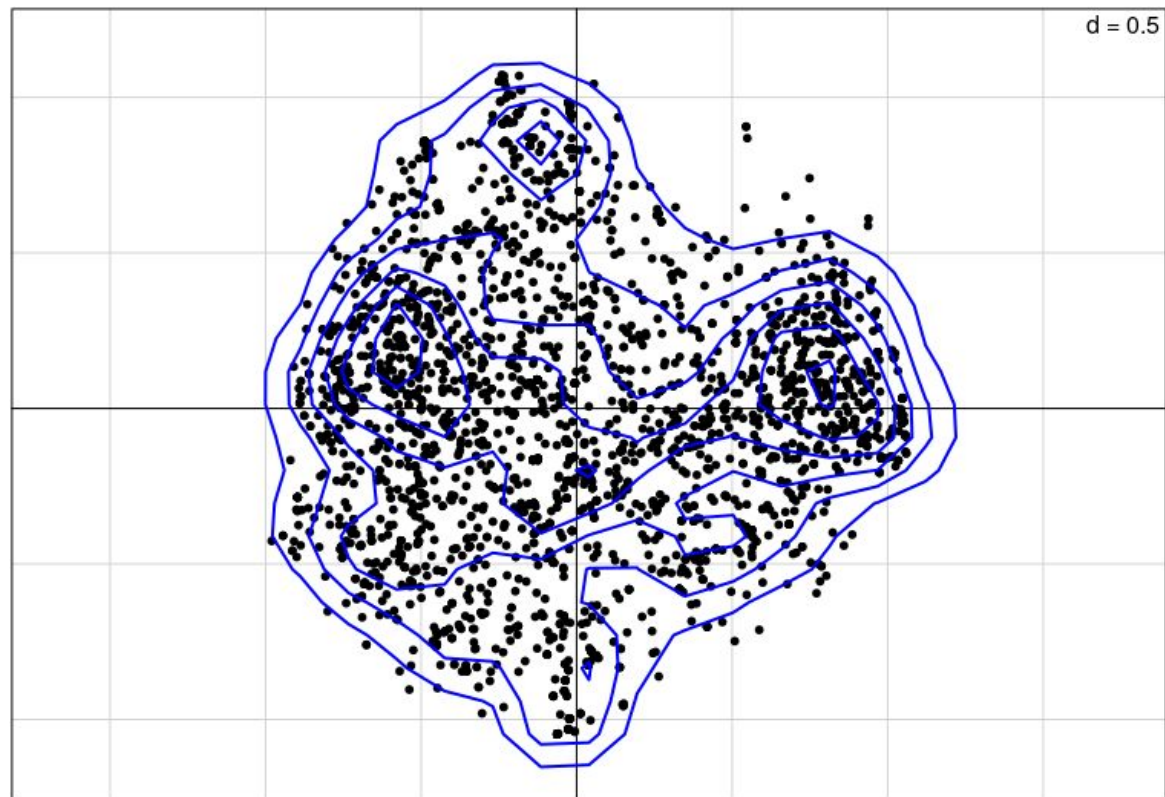

Fig S5: PCoA scatter plot with kernel density estimation

Supplement: FIG S5 [file mBio.00392-19-sf005.pdf]

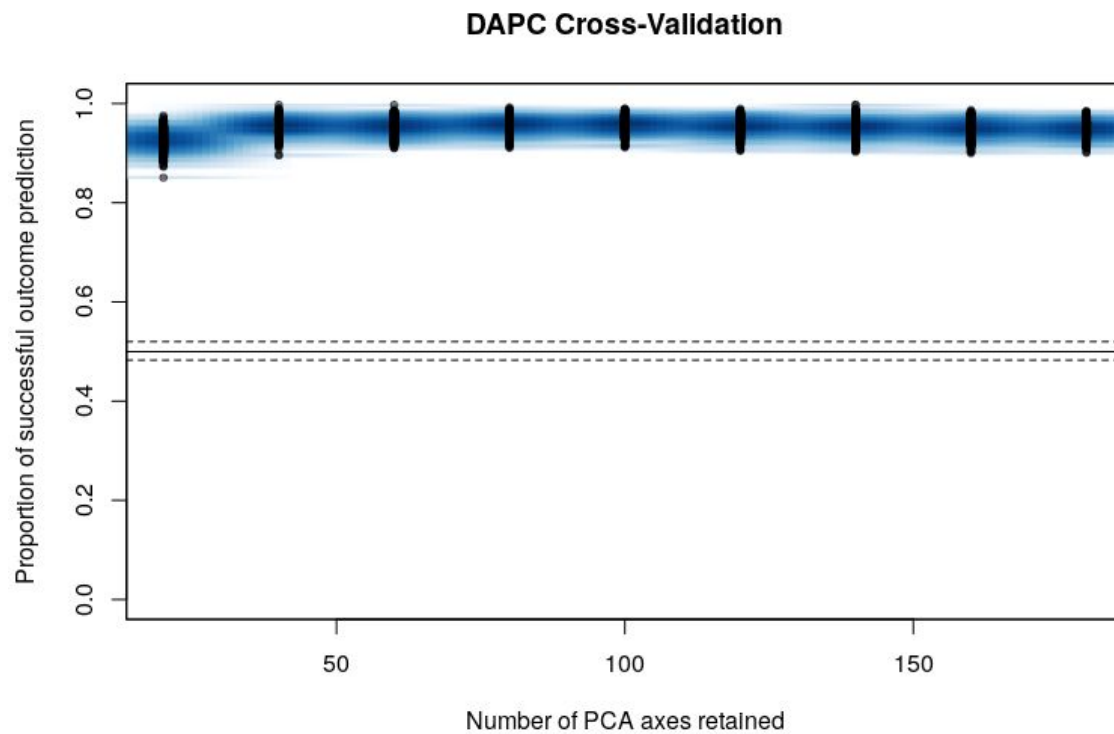

Fig S6: DAPC cross-validation. Optimal number of PCs is 100.

Supplement: FIG S6 [file mBio.00392-19-sf006.pdf]

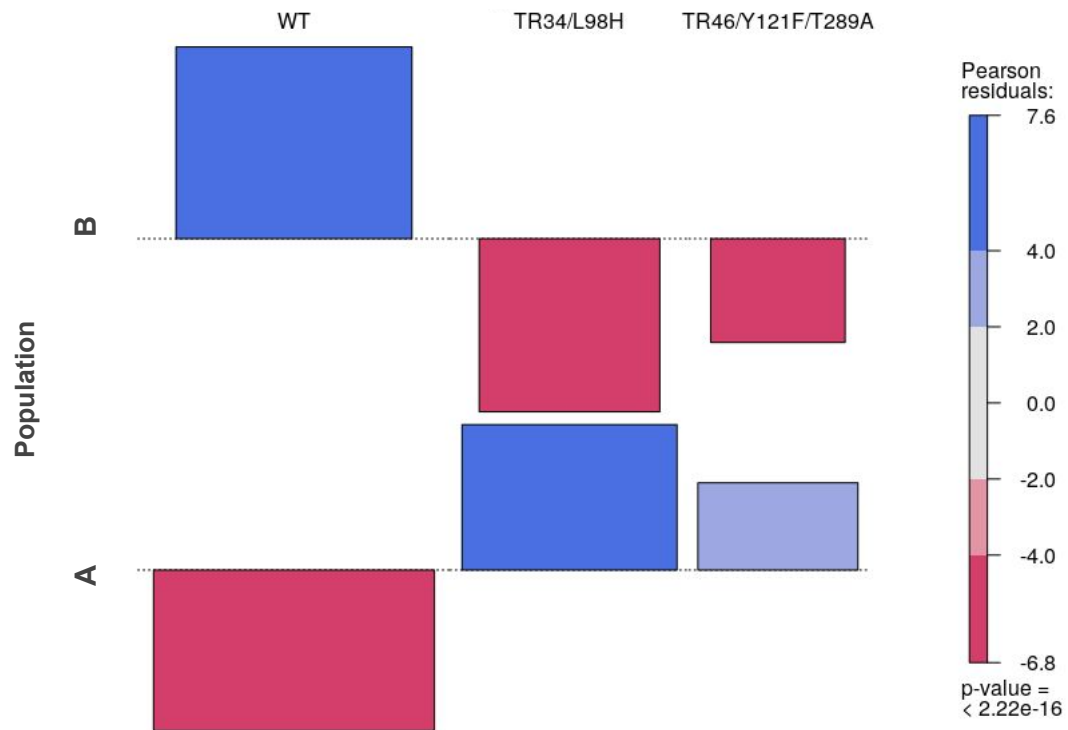

Fig S7: Association (mosaic) plot

Supplement: FIG S7 [file mBio.00392-19-sf007.pdf]
