# Supplementary material for: Nonrandom Distribution of Azole Resistance across the Global Population of Aspergillus fumigatus
Source: mBio. 2019 May 21;10(3):e00392-19. doi: 10.1128/mBio.00392-19 (PMC6529631; doi:10.1128/mBio.00392-19)
Supplement: FIG S2 [file mBio.00392-19-sf002.pdf]

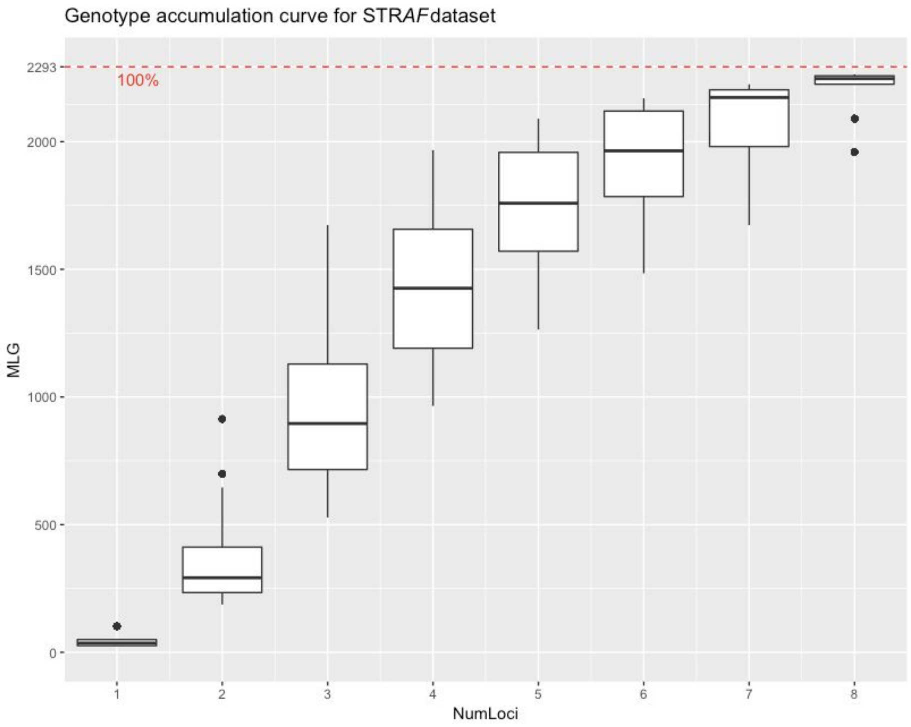

Fig S2: Genotype accumulation curve. Distributions generated using random sampling of loci 1000 times.
