# Supplementary material for: Nonrandom Distribution of Azole Resistance across the Global Population of Aspergillus fumigatus
Source: mBio. 2019 May 21;10(3):e00392-19. doi: 10.1128/mBio.00392-19 (PMC6529631; doi:10.1128/mBio.00392-19)
Supplement: FIG S3 [file mBio.00392-19-sf003.pdf]

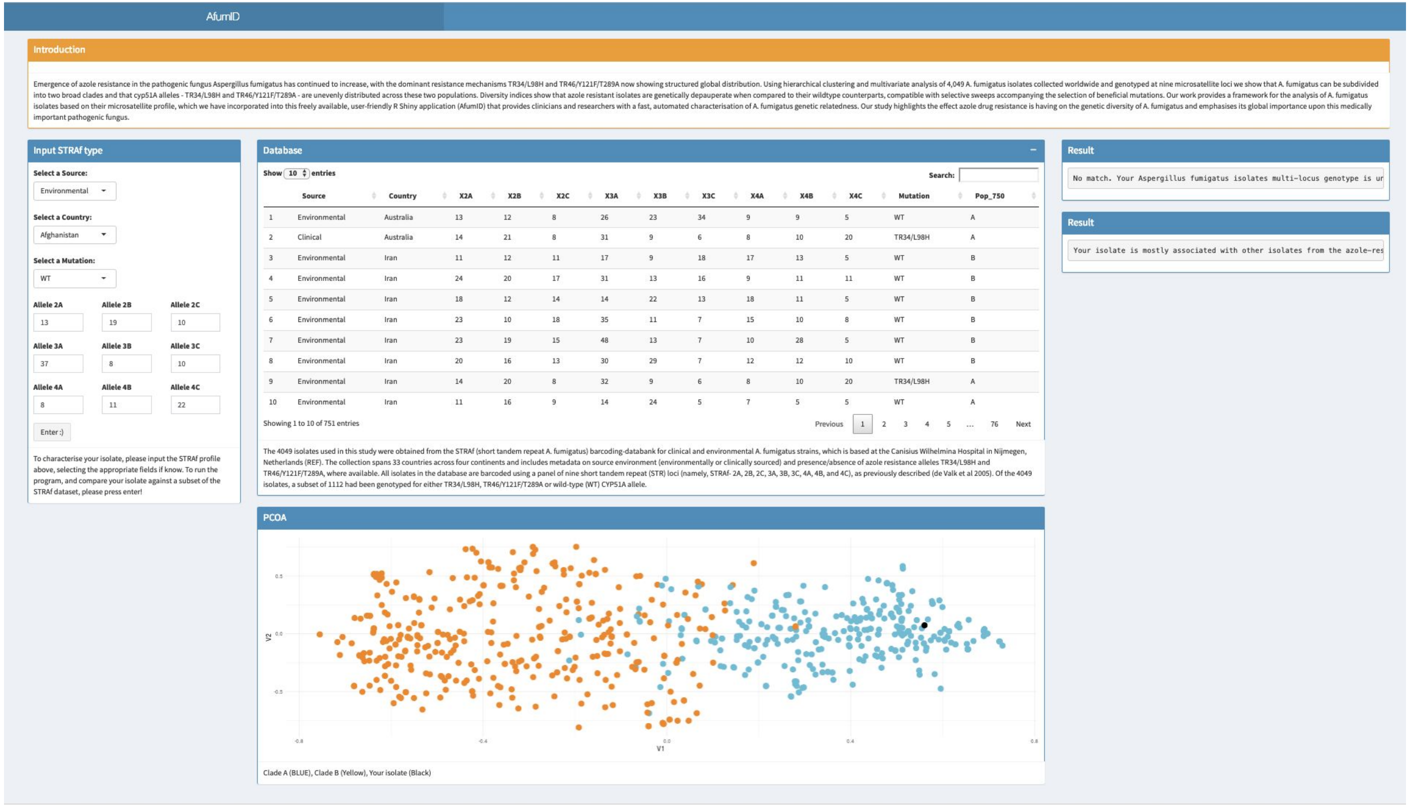

Fig S3: Screenshot of AfumID developed using R-Shiny. Accessible at https://afumid.shinyapps.io/afumID/
