# Supplementary material for: Nonrandom Distribution of Azole Resistance across the Global Population of Aspergillus fumigatus
Source: mBio. 2019 May 21;10(3):e00392-19. doi: 10.1128/mBio.00392-19 (PMC6529631; doi:10.1128/mBio.00392-19)
Supplement: FIG S4 [file mBio.00392-19-sf004.pdf]

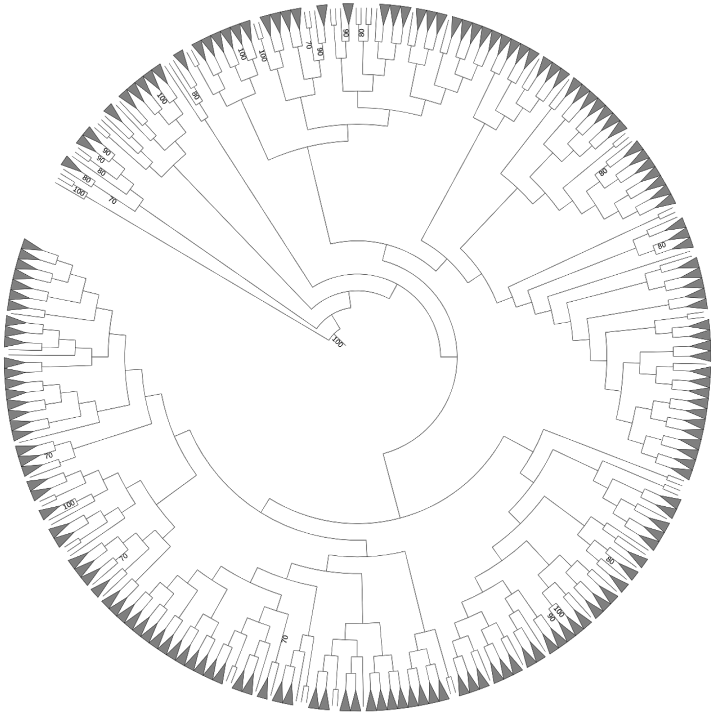

Fig S4: Dendrogram based on Bruvo genetic distance. Collapsed clades represent individuals with an average branch length of < 0.2 Bruvo’s distance.
