# Supplementary material for: Nonrandom Distribution of Azole Resistance across the Global Population of Aspergillus fumigatus
Source: mBio. 2019 May 21;10(3):e00392-19. doi: 10.1128/mBio.00392-19 (PMC6529631; doi:10.1128/mBio.00392-19)
Supplement: FIG S8 [file mBio.00392-19-sf008.pdf]

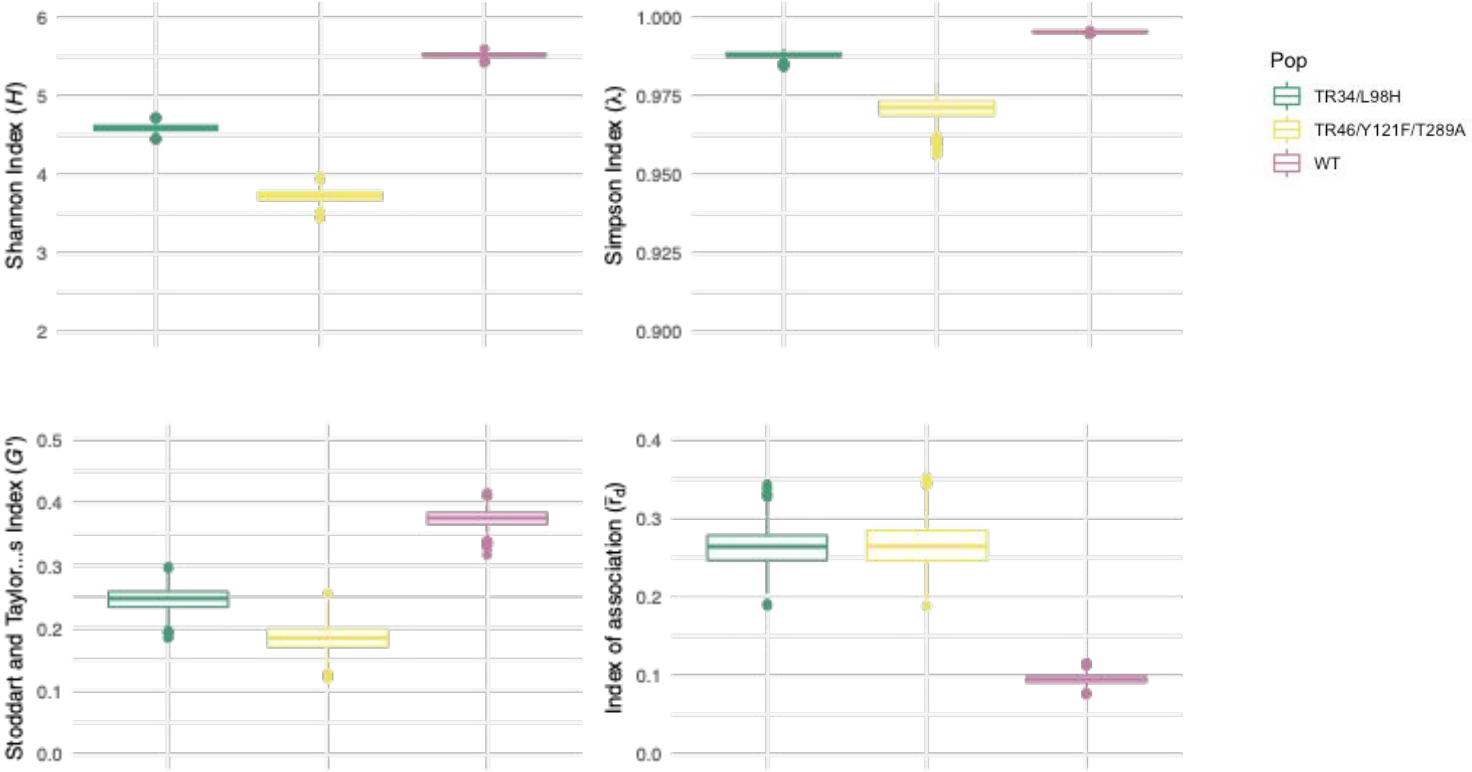

Fig S8: Clone-corrected genetic diversity indices of resistant Aspergillus fumigatus isolates harbouring either WT, TR34/L98H or TR46/Y121F/T289A CYP51A alleles.
