# Supplementary material for: Nonrandom Distribution of Azole Resistance across the Global Population of Aspergillus fumigatus
Source: mBio. 2019 May 21;10(3):e00392-19. doi: 10.1128/mBio.00392-19 (PMC6529631; doi:10.1128/mBio.00392-19)
Supplement: FIG S9 [file mBio.00392-19-sf009.pdf]

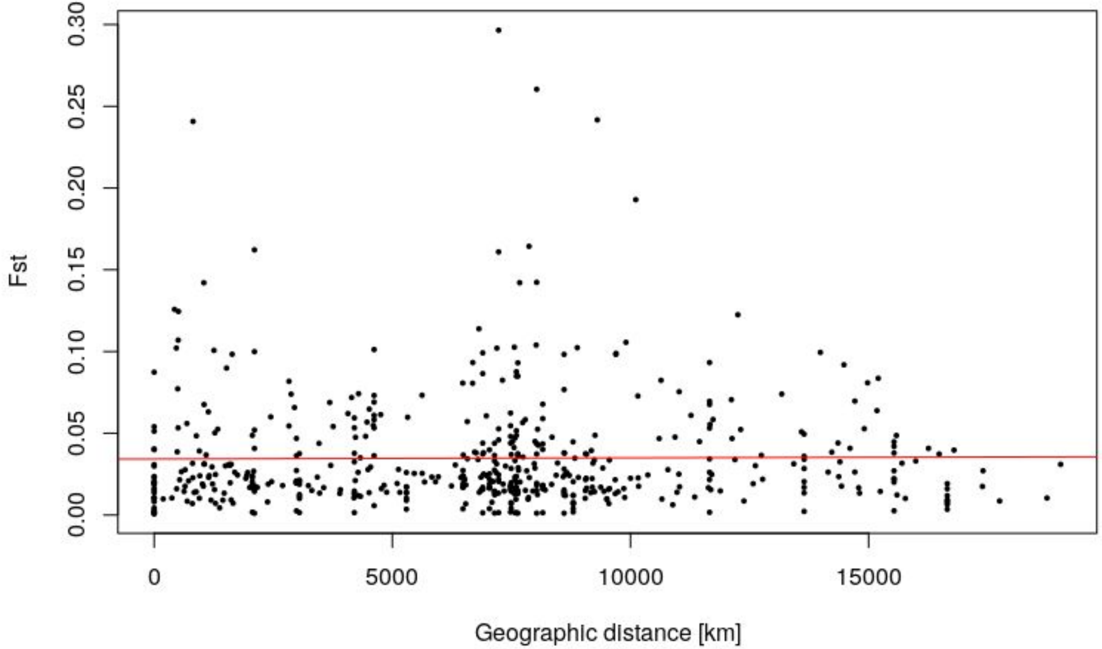

Fig S9: Isolation by distance scatter plot illustrating the pairwise relationship between genetic distance (country based Fst) and geographic distances (km).
